# Proinflammatory Cytokines in Women with PCOS in Atypical Pathogen Infections

**DOI:** 10.3390/diagnostics15131669

**Published:** 2025-06-30

**Authors:** Izabela Chudzicka-Strugała, Iwona Gołębiewska, Grzegorz Brudecki, Wael Elamin, Beata Banaszewska, Marta Chudzicka-Adamczak, Dominik Strugała, Barbara Zwoździak

**Affiliations:** 1 Department of Medical Microbiology, Poznan University of Medical Sciences, Rokietnicka 10, 60-806 Poznan, Poland; ichudzicka@vp.pl (I.C.-S.); barbara.zwozdziak@gmail.com (B.Z.); 2 Faculty of Medicine, Prince Mieszko I Medical University of Applied Sciences in Poznań, Bulgarska 55, 60-321 Poznan, Poland; 3 M42 (Healthcare), Masdar City, Abu Dhabi P.O. Box 4200, United Arab Emirates; 4 Department of Laboratory Diagnostics, Poznan University of Medical Sciences, Szamarzewskiego 82/84, 60-569 Poznan, Poland; 5 Department of Mechanical Engineering, Stanisław Staszic State University of Applied Sciences, 64-920 Piła, Poland; 6 Institute of Informatics and Quantitative Economics, Poznań University of Economics and Business, Towarowa 55, 60-995 Poznan, Poland

**Keywords:** PCOS, infertility, *Chlamydia trachomatis*, *Mycoplasma hominis*, *Ureaplasma* spp., *Ureaplasma urealyticum*, *Ureaplasma parvum*, atypical pathogen infections, IL-1β, Il-6, TNF-α, inflammation of the genital tract, BV, vaginal dysbiosis, vaginal microbiome, cytokines

## Abstract

**Background/Objectives:** Polycystic ovary syndrome (PCOS) is one of the most frequently diagnosed endocrine and metabolic disorders in women of reproductive age before menopause. It is associated with excess androgens and ovarian dysfunction, reduced fertility, the presence of obstetric disorders, but also metabolic disorders, and, among others, insulin resistance, obesity and type II diabetes. Its close relationship with changes in the diversity of the vaginal microbiome, vaginal inflammation and changes in the vaginal microenvironment, which can pave the way for pathogenic microorganisms, is emphasized. **Methods:** The research in the presented paper focuses on a group of women with PCOS (*n* = 490) of reproductive age (26–43 years), in whom the frequency of infections of the reproductive system caused by atypical pathogens, *Chlamydia trachomatis*, *Mycoplasma hominis* and *Ureaplasma* spp., were analyzed, and then the immune system response was assessed in terms of the level of serum proinflammatory cytokines, IL-1β, IL-6 and TNF-α. **Results:** Our results showed a 40% infection rate in the studied group of patients with PCOS, with *C. trachomatis* being the most common pathogen (17.7%), followed by *Ureaplasma* spp. (10%) and *M. hominis* (4.9%). In some cases, co-infections such as Mycoplasma and Ureaplasma were also observed in 3.1% or all three atypical bacteria, *M. hominis*, *Ureaplasma* spp. and *C. trachomatis*, in 4.3% of patients with PCOS. In our study, in women with PCOS and confirmed infection with any atypical pathogen (*n* = 196), we analyzed the levels of proinflammatory cytokines, IL-1 β a, IL-6 and TNF-α. The results were compared with a control group (control group A) consisting of patients with the same underlying disease, i.e., PCOS (*n* = 39), who did not experience infection with atypical pathogens or symptoms of gynecological infection. Additionally, a control group B (*n* = 28) consisting of healthy women (without PCOS and without infection) was introduced. The results regarding the levels of cytokines studied in this work (IL-1β, IL-6, TNF-α) may suggest that the presence of intracellular *C. trachomatis* in the infection will play a dominant role in the immune system response. In the infections with atypical pathogens analyzed in this study in patients with PCOS, no characteristic clinical features were observed, apart from indications in the form of an increase in the number of leukocytes in the assessment of the vaginal biocenosis, suggesting cervicitis and reported reproductive failure or lower abdominal pain. An additional problem is the inability to detect the presence of atypical pathogens in routine microbiological tests; therefore, confirmation of such etiology requires referral of the patient for targeted tests. **Conclusions:** Invasion of host cells by atypical pathogens such as C. trachomatis and infections with “genital mycoplasmas” can disrupt the function of these cells and lead to many complications, including infertility. The immune response with the production of proinflammatory cytokines such as TNF-α, IL-1β, and IL-6, observed in response to infection with C. trachomatis, M. hominis, and Ureaplasma spp., induces or amplifies inflammation by activating immune cells or controlling infection, but may lead to the facilitation of the survival of pathogenic microorganisms and irreversible damage to fallopian tube tissues. Especially in the case of the proinflammatory cytosine TNF-α, there seems to be a close correlation with infections with atypical pathogens and a marked immune response, as well as with increased IL-1β and IL-6 values compared with the absence of infection (both in the presence and absence of PCOS). The presented study may suggest the importance of extended diagnostics to include atypical pathogens in the case of PCOS and the importance of research in this area also from the point of view of the immune response.

## 1. Introduction

Polycystic ovary syndrome (PCOS) is one of the most frequently diagnosed endocrine and metabolic disorders in premenopausal women of reproductive age. It is associated with androgen excess and ovarian dysfunction, but its heterogeneous nature and complexity make it difficult to understand its direct etiology and the possibility of developing complications, including reduced fertility, obstetrical disorders, atypia or endometrial cancer, but also metabolic disorders, insulin resistance, obesity, type II diabetes, hypertension, vascular thrombosis, cerebrovascular accidents or mood disorders [1,2]. It has been suggested that PCOS is the most common cause of infertility in women of reproductive age [3,4]. Moreover, in women with PCOS, attention is paid to changes in the composition of the microbiome, resulting in dysbiosis and elimination of *Lactobacillus* spp. (e.g., *L. crispatus*) and an increased share of pathogenic microorganisms such as *Chlamydia trachomatis*, *Mycoplasma hominis*, *Mycoplasma genitalium*, *Prevotella* or *Gardnerella vaginalis* [5,6] as well as a predisposition to sexually transmitted infections [7,8]. In the course of PCOS, its close relationship with changes in the diversity of the vaginal microbiome and accompanying vaginitis [9] correlation with disturbed menstrual cycles and hormone levels is emphasized [10] Hence, the research in the presented work focused on a group of women with PCOS of reproductive age.

In addition, the association of endocrine disorders and immune dysregulation, potentially contributing to increased susceptibility to the development of sexually transmitted diseases, is emphasized during PCOS [11]. Disturbed cytokine production, immune cell dysfunction and hormonal abnormalities in PCOS result in chronic low-grade inflammation due to the accumulation of numerous inflammatory cells and many inflammatory cytokines [12,13,14].

The mechanisms of infectious processes, especially in the case of “atypical” pathogens, may additionally exacerbate and possibly accelerate pathological changes in the reproductive system of women with PCOS, promote chronic infections, limit reproductive functions and result in complications in the reproductive system. Therefore, the aim of our study was to analyze the incidence of reproductive system infections with the most common “atypical” pathogens, *C. trachomatis* and genital *Mycoplasma* spp. and *Ureaplasma* spp., in patients with PCOS of reproductive age in 2019–2023 with the assessment of proinflammatory cytokines such as interleukin-1β (Il-1β), interleukin-6 (IL-6) and tumor necrosis factor-alpha (TNF-α).

### 1.1. Atypical Infections

Infections with *C. trachomatis* serotypes D-K (chlamydiosis), according to the WHO, are one of the most common sexually transmitted diseases and a significant public health problem. Due to the nonspecific, mildly symptomatic or even asymptomatic clinical course of this disease entity, it may remain undiagnosed for a long time, resulting in infertility and increased susceptibility to other sexually transmitted infections. Therefore, appropriate microbiological diagnostics play a key role [15], especially diagnostic tests based on molecular biology, which, however, remain expensive and unavailable in every laboratory [16,17]. A similar situation is observed in the case of genital infections with *Mycoplasma hominis* and *Ureaplasma* spp. (*U. urealyticum*, *U. parvum*) because, although they are also common “atypical” pathogens, they are omitted in routine gynecological examinations and often remain undiagnosed [18,19].

*C. trachomatis* is an obligate intracellular bacterium with a unique development cycle, defining its atypicality. The infectious extracellular form is an elementary body that penetrates the cells of the mucosa, differentiating into intracellular reticular bodies, forming inclusions. Within the *C. trachomatis* species, there are three biotypes, with the genital biotype including serotypes D-K, which are sexually transmitted. It is estimated that infection with these serotypes is largely asymptomatic (70–80%) or has only mild symptoms, but in 15–40% of patients, it can lead to serious complications such as pelvic inflammatory disease (PID), infertility and ectopic pregnancy [20]. Untreated infections can facilitate the acquisition of infections such as HIV and other sexually transmitted diseases, hence, it is a global public health problem [17]. Additionally, chronic *C. trachomatis* D-K infections result in fibrosis and scarring of tissues in infected organs. Obstruction of fallopian tubes is observed after a single episode of their inflammation caused by *C. trachomatis*, and on average one in ten patients becomes infertile. With the increase in the number of *C. trachomatis* infections, an increase in infertility cases is observed at the level of 35–70% [17], and the risk of PID (symptomatic or asymptomatic) is 17.1% [21].

Mycoplasmas, on the other hand, are the smallest free-living bacteria (0.15–0.30 μm) with reduced metabolic pathways, whose taxonomic classification is evolving [22]. Although colloquially, also among clinicians, “genital mycoplasmas” include, among others, *M. hominis*, *Ureaplasma* spp. (*U. urealyticum*, *U. parvum*). Their “atypicality” is mainly related to the lack of a cell wall and special growth requirements [23]. Hence, these microorganisms are also not commonly detected in routine microbiological tests, and specialized tests include directed culture or molecular biology NAAT [24,25,26]. *Mycoplasma* spp. are extracellular parasites for which adhesion is an essential virulence factor in disease pathogenesis [27]. Although there are situations in which genital *Mycoplasma* spp. enter non-phagocytic host cells [28], the contribution to pathobiological changes in the host cell is still not fully understood [29]. The cause of chronic *Mycoplasma* spp. infections a believed to be molecular mimicry and phenotypic plasticity, resulting in impaired recognition by the host immune system. The lack of a cell wall provides phenotypic diversity [27,28,30], facilitating the survival of the immune response, providing a “good fit” or “glue” to host tissue receptors and facilitating colonization [27,31,32,33].

Due to the significant involvement of *Mycoplasma* spp. in the etiology of sexually transmitted diseases and their ability to cause acute and chronic infections of the genitourinary system, as well as their opportunistic nature, in recent years, there has been an increased interest in these microorganisms [34,35], as well as in species of the genus Ureaplasma. These pathogens also play an important role in the development of gynecological, obstetrical and urological diseases and many complications, both in pregnant women and newborns [29,36].

### 1.2. Cytokines Response

Cytokines play an important role in regulating the immune and inflammatory response, controlling the duration but also the strength of the immune response. Many diseases associated with PCOS, such as diabetes and obesity, are believed to affect the abnormal production of cytokines, which leads to ovulatory dysfunction [37]. Numerous mediators, including proinflammatory cytokines such as tumor necrosis factor (TNF)-α, interleukin IL-1β and IL-6, regulate inflammation [38]. Sex hormones have an important regulatory function in the production of many proinflammatory cytokines, including IL-6 [39]. Moreover, the key role of cytokines such as TNF-α and IL-6 is emphasized in the process of ovarian steroidogenesis, apoptosis of granulosa and luteal cells, follicular atresia and even in the impact on oocyte quality [37]. In infections with “atypical” pathogens, such as *C. trachomatis*, an important role is played by the activation of the immune response with the production of cytokines, including interleukin-1 (IL-1), interleukin-6 (IL-6) and tumor necrosis factor-alpha (TNF-α). These factors are proinflammatory and, on the one hand, may be helpful in limiting the infection, but they also participate in irreversible tissue damage due to the persistent survival of pathogens in the host organism [40]. Hence, the importance of *C. trachomatis* in chronic infections, as well as the induction of pathological processes, is emphasized. An example is the involvement of, for example, IL-1, leading to damage to the fallopian tubes. In turn, infection of trophoblast cells by *C. trachomatis* causes an increase in the concentration of interleukin-1β (IL-1β) [41]. Increased levels of proinflammatory cytokines such as IL-6 and IL-1β are also observed in *Mycoplasma* spp. infections [42], and *Ureaplasma* spp. may direct monocyte immune responses towards proinflammatory responses, with immunomodulatory features being indicated in the presence of another pathogen. *Ureaplasma* spp., in the presence of an additional pathogen, may attenuate the proinflammatory cytokine response, resulting in the inhibition of effective immune defense and persistence of chronic infections [43], characteristic of “atypical” pathogens.

## 2. Materials and Methods

The aim of the study presented here was to assess the incidence of infections with so-called atypical pathogens, which are not detected in routine microbiological examinations of the genital tract, *C. trachomatis*, *M. hominis* and *Ureaplasma* spp. (*U. urealyticum/U. parvum*), and to assess the immune response to proinflammatory cytokines important in infections with atypical pathogens, such as interleukin-1β (IL-1β), interleukin-6 (IL-6) and tumor necrosis factor-alpha (TNF-α), in patients of reproductive age with PCOS.

### 2.1. Patient Group

The present study included 490 women (aged 26–43 years) with PCOS diagnosed according to the Rotterdam Consensus criteria for PCOS and presenting at least two of the following symptoms: (1) oligo- or amenorrhea; (2) clinical or chemical hyperandrogenism and/or (3) polycystic ovaries determined by transvaginal ultrasound examination [44]. In the analyzed patients with diagnosed PCOS, the following were excluded: thyroid disease, Cushing’s disease, diabetes, hyperprolactinemia and congenital adrenal hyperplasia. The patients were sexually active, had not taken any contraceptives for at least a year and had no infections such as gonorrhea, trichomoniasis, syphilis or HIV. The patients were referred to a gynecologist for check-ups before a planned pregnancy, for gynecological examinations without complaints or in the case of reproductive failure, lower abdominal pain or suggestion in the microbiological examination of the vaginal biocenosis due to a high number of leukocytes in the analysis. Patients were referred for further evaluation because leukorrhea, defined as >10 WBCs/HPF on microscopic examination of vaginal discharge, is considered an indicator of cervicitis [45,46,47]. Microbiological tests for “atypical” pathogens such as *C. trachomatis*, *M. hominis* and *Uraeplasma* spp. (*U. urealyticum*/*U. parvum*) were performed in all patients.

In addition, due to the possibility of elevated cytokine levels in the case of the underlying disease, i.e., polycystic ovary syndrome in women, two control groups were selected. The control group (group A) consisted of 39 patients diagnosed with PCOS, without any complaints and with a normal assessment of the vaginal biocenosis, in whom microbiological tests for the presence of “atypical” pathogens gave negative results. In order to compare the levels of selected cytokines with the levels in healthy women, the control tests were extended to include a group (group B; *n* = 28) of patients without PCOS and without any complaints, without infections, with a normal assessment of the vaginal biocenosis and negative results of microbiological tests performed for the presence of “atypical” pathogens.

### 2.2. Material

The diagnostic material for microbiological tests to detect “atypical” pathogens was a swab from the cervical canal (*C. trachomatis*) and a swab from the posterior vaginal fornix (*M. hominis*, *Ureaplasma* spp.).

The assessment of the concentration of selected cytokines, IL-1β, IL-6 and TNF-α, was performed in serum from collected venous blood. Serum from patients with PCOS, in whom infections with any of the “atypical” pathogens were detected, and from both control groups, control group A—patients with PCOS without infection with an atypical pathogen, and control group B—patients without PCOS and without infection with an atypical pathogen, was tested. The study was approved by the Institutional Evaluation Committees at the Poznan University of Medical Sciences (no. 1134/16).

### 2.3. Methods

The presence of *C. trachomatis* in the tested biological material (cervical swab) was detected using the qualitative PCR method (Abbott RealTime CT/NG test) [48,49]. *M. hominis*, *Ureaplasma* spp. (in the swab from the posterior vaginal fornix) were detected using the MYCOPLASMA IST3 test (Biomerieux, Marcy-l′Étoile, France) [50]. This test was selected due to the possibility of obtaining simultaneous results in terms of drug susceptibility for the tested pathogens.

In the case of positive results for the presence of any of the analyzed “atypical” pathogens and in all uninfected women in the control groups, group A (with PCOS) and group B (without PCOS), the levels of the following cytokines IL-1β, IL-6 and TNF-α were tested. All tests for selected proinflammatory cytokines were performed in serum using the immunoenzymatic ELISA method (Enzyme Linked Immunosorbent Assay) using kits: IL-1β—Quantikine HS ELISA Human IL-1β/IL-1F2 Immunoassay (R&D Systems, Minneapolis, MN, USA), IL-6—Quantikine ELISA Human IL-6 Immunoassay (R&D Systems) and TNF-α tests—Quantikine ELISA Human TNF-α Immunoassay (R&D Systems). Absorbance was read in a Reader 250 spectrophotometer (bioMerieux) at a wavelength of λ = 450 nm. Results were calculated based on established standard curves for each cytokine analyzed.

## 3. Results

### 3.1. Assessment of the Prevalence of Atypical Pathogen Infections in a Study Population of Women with PCOS

**Figure 1:**
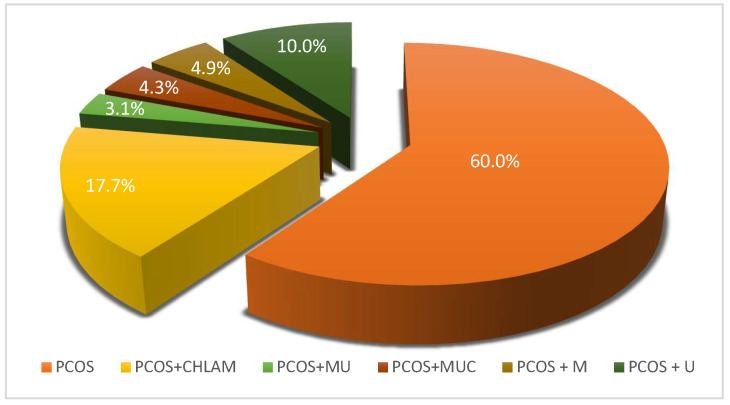
Percentage of atypical pathogens infections in the studied PCOS patients (*n* = 490). PCOS—patients with PCOS and without atypical pathogen infection (*n* = 294; 60%). Infections with atypical pathogens in patients with PCOS (*n* = 196; 40%) were divided as follows: PCOS + CHLAM—patients with PCOS and *C. trachomatis* infection (*n* = 87; 17.7%); PCOS + MU—patients with PCOS, *M. hominis* and *Ureaplasma* spp. (*U. urealyticum*/*U. parvum*) infection (*n* = 15; 3.1%); PCOS + MUC—patients with PCOS, *C. trachomatis*, *M. hominis* and *Ureaplasma* spp. (*U. urealyticum*/*U. parvum*) infection (*n* = 21; 4.3%); PCOS + M—patients with PCOS and *M. hominis* infection (*n* = 24; 4.9%); PCOS + U—patients with PCOS and *Ureaplasma* spp. (*U. urealyticum*/*U. parvum*) infection (*n* = 49; 10%).

In this study, the frequency of infections with atypical pathogens, *C. trachomatis*, *M. hominis* and *Ureaplasma* spp. (*U. urealyticum*, *U. parvum*), was analyzed in the study group (*n* = 490) of reproductive age (26–43 years) diagnosed with PCOS. The occurrence of any atypical pathogens in the study group of patients with PCOS was detected in 40% (*n* = 196). Among the atypical pathogens, *C. trachomatis* was detected in 87 (17.7%) patients with PCOS, *Ureaplasma* spp. infection in 49 (10%) women and *Mycoplasma* spp. in 24 (4.9%) cases. Coinfection was detected in 15 (3.1%) patients with PCOS for Mycoplasma and Ureaplasma, and simultaneous infection with all three bacteria, *M. hominis*, *Ureaplasma* spp. and *C. trachomatis* in 21 (4.3%) cases. The frequency of infections with atypical pathogens without a cell wall—so-called genital mycoplasmas, i.e., *M. hominis/Ureaplasma* spp. combined (*n* = 88)—was 18%. Hence, the incidence in the entire studied group of PCOS patients (*n* = 490) for intracellular *C. trachomatis* and extracellular, without cell wall—genital Mycoplasma and Ureaplasma—is about 18% in both cases. (Figure 1). In this study, we did not detect separately distinguished species for the genera Mycoplasma and Ureaplasma due to the limitations of the applied test. Therefore, the result for Mycoplasma was representative of *M. hominis* and *Ureaplasma* spp. (*U. urealyticum*/*U. parvum)*.

### 3.2. Evaluation of Cytokine (TNF-α, IL-1β and IL-6) Levels in a Study Population of Women with PCOS

In our study, in women with PCOS and confirmed infection with any atypical pathogen (*n* = 196), we analyzed the levels of proinflammatory cytokines, such as IL-1β, IL-6 and TNF-α, which are important in this type of infection (Table 1). The results were compared with the control group (control group A) consisting of patients with the same underlying disease, i.e., PCOS (*n* = 39), who did not have infections with atypical pathogens or symptoms of a gynecological infection.

The table below (Table 1) presents the mean values of the concentrations of the analyzed proinflammatory cytokines; TNF-α, IL-1β and IL-6 (expressed in picograms per milliliter [pg/mL]) ± standard deviation measured in patients with PCOS with infections caused by atypical pathogens and in the control groups. The results were grouped according to infections as follows: *C. trachomatis* infections, infections caused by “genital mycoplasmas” (*M. hominis* and *Ureaplasma urealyticum/U. parvum*) collectively, i.e., the occurrence separately and in co-infection was included together, and the third group consisted of simultaneous infection with *C. trachomatis* as well as *M. hominis* and *Ureaplasma* spp. *(U. urealyticum/U. parvum*).

**Table 1:** Mean concentration values [pg/mL] ± standard deviation of selected proinflammatory cytokines: TNF-alpha, IL-1β and IL-6 in infections with atypical pathogens in patients with PCOS. CHLAM—patients with PCOS and *C. trachomatis* infection; MU—patients with PCOS and *M. hominis*, *Ureaplasma* spp. (*U. urealyticum*/*U. parvum*) infection (separately and in co-infection); MUC—patients with PCOS, *C. trachomatis*, *M. hominis* and *Ureaplasma* spp. (*U. urealyticum*/*U. parvum*) infection; control group A—patients with PCOS without infections; control group B—healthy patients (without PCOS and infections).

	CHLAM (*n* = 87)	MU (*n* = 88)	MUC (*n* = 21)	Control Group A	Control Group B
TNF α [pg/mL]	26.27 ± 6.50	22.16 ± 5.29	29.90 ± 7.32	8.02 ± 3.53	5.24 ± 1.30
IL-1 β [pg/mL]	3.07 ± 0.82	2.47 ± 0.73	3.56 ± 1.25	1.33 ± 0.39	1.14 ± 0.18
IL-6 [pg/mL]	2.77 ± 0.80	2.39 ± 0.62	3.27 ± 1.04	1.61 ± 0.41	1.25 ± 0.31

**Figure 2:**
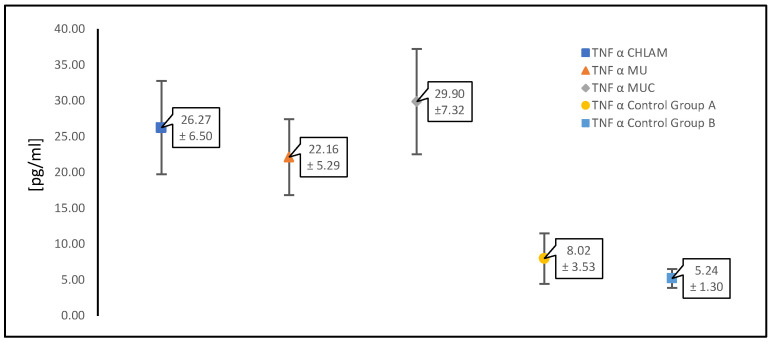
Summary of the results of the study of the proinflammatory cytokine TNF-α in PCOS patients infected with an atypical pathogen (divided according to the occurrence of pathogens) compared to the control groups A and B. TNF-α CHLAM—patients with PCOS and *C. trachomatis* infection; TNF-α MU—patients with PCOS, *M. hominis* and *Ureaplasma* spp. (*U. urealyticum*/*U. parvum*) infection; TNF-α MUC—patients with PCOS, *C. trachomatis*, *M. hominis* and *Ureaplasma* spp. (*U. urealyticum*/*U. parvum*) infection; TNF-α control group A—patients with PCOS without infections; TNF-α control group B—patients without PCOS and without infections.

**Figure 3:**
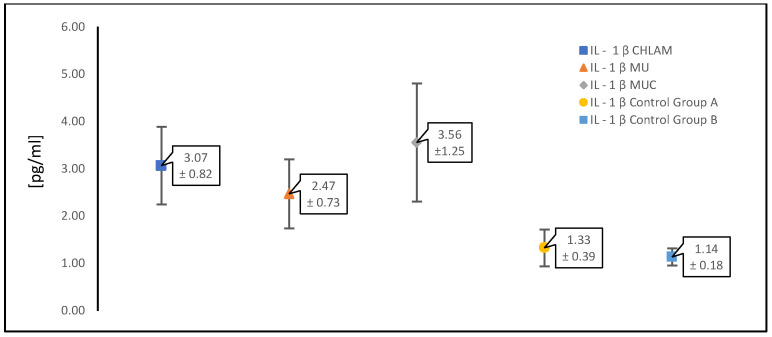
Summary of the results of the study of the proinflammatory cytokine IL-1β in PCOS patients infected with an atypical pathogen (divided according to the occurrence of pathogens) compared to the control groups A and B. IL-1β CHLAM—patients with PCOS and *C. trachomatis* infection; IL-1β MU—patients with PCOS, *M. hominis* and *Ureaplasma* spp. (*U. urealyticum*/*U. parvum*) infection; IL-1β MUC—patients with PCOS, *C. trachomatis*, *M. hominis* and *Ureaplasma* spp. (*U. urealyticum*/*U. parvum*) infection; IL-1β control group A—patients with PCOS without infections; IL-1β control group B—patients without PCOS and without infections.

**Figure 4:**
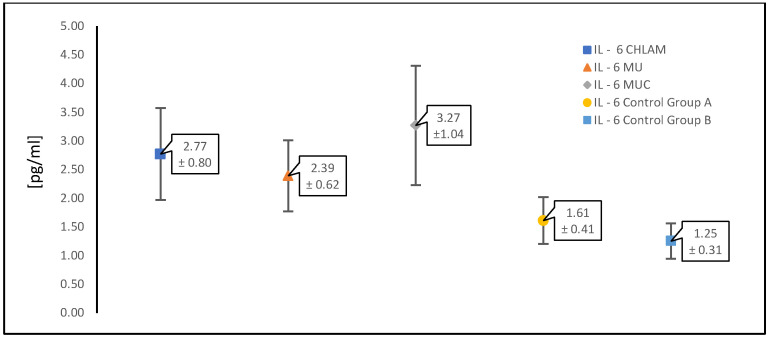
Summary of the results of the study of the proinflammatory cytokine IL-6 in PCOS patients infected with an atypical pathogen (divided according to the occurrence of pathogens) compared to the control groups A and B. IL-6 CHLAM—patients with PCOS and *C. trachomatis* infection; IL-6 MU—patients with PCOS, *M. hominis* and *Ureaplasma* spp. (*U. urealyticum*/*U. parvum*) infection; IL-6 MUC—patients with PCOS, *C. trachomatis*, *M. hominis* and *Ureaplasma* spp. (*U. urealyticum*/*U. parvum*) infection; IL-6 control group A—patients with PCOS without infections; IL-6 control group B—patients without PCOS and without infections.

Figure 2, Figure 3 and Figure 4 show scatter plots illustrating mean concentrations [pg/mL] ± standard deviation. In each figure presented (Figure 2, Figure 3 and Figure 4), the points on the graphs represent the mean concentration values for each specified group, and the error bars show the standard deviation, reflecting the variability within the groups. The results on the graphs are presented for each of the cytokines analyzed first as a pooled comparison between the groups, and then as individual pairwise comparisons between the two control groups and between atypical intracellular pathogens and pathogens without cell wall infection types for each group.

Figure 2 shows a comparison between groups of patients infected with atypical pathogens in individual combinations and the control groups A and B for the cytokine TNF-α. The next ones are in the same arrangement for the cytokine IL-1β (Figure 3) and the cytokine IL-6 (Figure 4).

In our study, Welch’s test was used to determine the significance of differences in the range of the analyzed proinflammatory cytokines, TNF-α, IL-1β and IL-6, associated with the three tested atypical pathogens and their co-infections. Welch’s test was used due to its flexibility in medical research, especially in the case of heterogeneous clinical data. Welch’s test was used to assess whether the variance of the immune response, associated with the levels of selected cytokines, differs significantly after exposure to different atypical pathogens. The performed Welch’s test compares the mean concentration values of two groups if their variances may differ. For each of the proinflammatory cytokines we analyzed, TNF-α (Table 2), IL-1β (Table 3), and IL-6 (Table 4), similar observations were found. Statistically significant differences (*p*-value < 0.05) were shown for: *C. trachomatis* infection compared to control group A, Mycoplasma/Ureaplasma infection compared to control group A, co-infection with three atypical pathogens (*C. trachomatis*, Mycoplasma, Ureaplasma) compared to control group A, *C. trachomatis* infection compared to Mycoplasma/Ureaplasma infection, Mycoplasma/Ureaplasma infection compared to co-infection with three atypical pathogens (*C. trachomatis*, *M. hominis*, *Ureaplasma* spp.) and the comparison between the two groups control A (women with PCOS without infection) with control B (women without PCOS and without infection). This may mean that when comparing patients with different infections, and one group has a significantly higher level of a specific biomarker, it suggests that a given infection affects that parameter and is not the result of random changes in the data.

However, no statistically significant difference (*p* > 0.05) was observed for any of the analyzed proinflammatory cytokines, TNF-α, IL-1β and IL-6, in the comparison of PCOS patients with *C. trachomatis* infection with PCOS patients and co-infection with three atypical pathogens, *C. trachomatis*, genital *Mycoplasma* spp. and *Ureaplasma* spp. This result could suggest that the presence of intracellular *C. trachomatis* in the infection will play a dominant role in the immune system response because the presence of an additional infection in the form of “genital mycoplasmas” does not influence the changes in the cytokine response, at least in the scope of the proinflammatory cytokines analyzed in this work.

Table 2 presents the concentration values for TNF-α, and the greatest differences can be observed in relation to the control groups. A relatively small increase in this proinflammatory cytokine is observed in the presence of PCOS syndrome alone compared to the group of healthy patients. However, regardless of the atypical pathogen, this increase is significant in each infection, although always the highest in the presence of *C. trachomatis* infection.

Table 3 and Table 4 present the results for the concentration of cytokines IL-1β and Il-6, respectively. In the case of infections with atypical pathogens, their concentration increases, but it is not as notable as for TNF-α (Table 2). Additionally, both cytokines do not show a significant increase in the presence of PCOS alone without infection (control group A) compared to the group of healthy women (control group B). Moreover, the greatest differences and the highest increase are observed, although they are not large, as in the case of TNF-α, in the presence of *C. trachomatis*.

## 4. Discussion

Sexually transmitted diseases (STDs) are a major public health problem worldwide. One of the most common pathogens is *C. trachomatis*. Increasing attention is also being paid to the involvement of urogenital *M. hominis* and *Ureaplasma* spp. (*U. parvum*, *U. urealyticum*), although their role as STDs is not yet clear. However, in the case of all these atypical pathogens, their importance in the development of infertility is indicated, most often as a result of persistent or unrecognized infections. That is, all those who are not treated for various reasons, e.g., due to lack of diagnosis or inadequate therapy [51,52]. In addition to infertility, the role of atypical pathogens is also important in the etiology of pregnancy complications, e.g., induction of preterm labor, miscarriages, pelvic inflammatory disease, but also infections and complications in the newborn, e.g., severe bronchopulmonary dysplasia, infections of the central nervous system (CNS) by *Ureaplasma* spp. or *Mycoplasma* spp. or atypical pneumonia and conjunctivitis caused by *C. trachomatis* [45,46,47,52]. Currently, data from scientific studies are not clear regarding the frequency of infections with atypical pathogens, some indicate a significant share of *Ureaplasma* spp. (~21%), and a lower share of *M. hominis* (3%) [53] and a similar level of *C. trachomatis* (~2.9%) in infections [48]. Other studies, including showed a high prevalence of *Ureaplasma* spp. and *M. hominis* (overall prevalence: 38.1%), with *Ureaplasma* spp. infections being more common (31.3%), *Ureaplasma* spp./*M. hominis* infections (6.0%) and *M. hominis* alone being the rarest (0.8%) [54]. Other authors also report the high prevalence of positive genital mycoplasmas in almost 34% in Beijing and 47% of patients in Xi’an (Wang et al., 2016; Zeng et al., 2016), as well as in studies from South Korea (Lee and Yang, 2020) and Romania (Doroftei et al., 2021) [49,50,55,56]. Some data indicate that in sexually mature, asymptomatic women, *Ureaplasma* spp. can be detected in up to 40–80%, *M. hominis* in 20–50%, while *M. genitalium* is the rarest and occurs in only 0–5% of them [57]. Data from different parts of the world are quite diverse, which of course may be related to different access to medical care and diagnostics, but also to the diagnostic methods used, as well as the population studied. A Czech study also showed a higher prevalence of *U. urealyticum* infection (39.6%) and *M. hominis* infection of 8.1% in patients undergoing initial fertility testing [58]. In a German study, *U. urealyticum* was predominant among infertile women tested for *U. urealyticum/U. parvum* (41.30%) and *M. hominis* (34.90%) [59], while the prevalence rates were lower for *U. urealyticum* 9.0% and *M. hominis* (8.6%) in women of reproductive age in the Italian study by [60,61,62,63,64]. These results are similar to the Brazilian study by Piscopo et al. (2020), which showed a lower rate of infections with *C. trachomatis* (3.7%), *U. urealyticum* (9.0%), *M. hominis* (5.7%), but they emphasized the importance of bacterial colonization of the cervix and the association between them and the development of infertility [53]. In another Brazilian study, in a population of infertile women, the prevalence of *C. trachomatis* was higher (10.9%) [65], and in asymptomatic European women), the prevalence of *C. trachomatis* ranged from 1.7% to 17% [54].

Thus, atypical pathogens undoubtedly play an important role in the development of infertility, but the indicated frequency of infections may be determined by many factors, including the population of patients studied. The results presented in our study do not differ from the global data, although the percentage of women diagnosed with infection with any of the atypical pathogens studied is quite high and constitutes 40% (*n* = 196) of the studied women with PCOS (*n* = 490). One may wonder whether this fact is caused by certain predispositions because our study focused on a group of women in whom PCOS disease may also predispose them to infections with such microorganisms, due to the state of the immune system and potential changes in the balance of the vaginal microbiome. In our study, *C. trachomatis* infections dominated, constituting 17.7% of all positive results, similar to other studies of patients, especially those with infertility. As a separate etiological factor, the second most common was *Ureaplasma* spp. (10%), and *Mycoplasma* spp. as a single etiological factor occurred the least frequently (4.9%). It should also be noted that some patients had mixed infections with “urogenital mycoplasmas”, i.e., *M. hominis* and *U. urealyticum* (3.1%), or even co-infections with all three atypical pathogens at the same time, i.e., *C. trachomatis*, *Ureaplasma* spp. and *Mycoplasma* spp. (4.3%). Of course, this has significant implications for the selection of appropriate treatment. The high percentage of women with PCOS and a positive result for *C. trachomatis*, but also for other atypical pathogens, can be explained by the narrow selection of the study group, which struggles with infertility, and the use of targeted microbiological diagnostics, which is not commonly performed in routine gynecological examinations.

Moreover, scientific studies indicate an association between PCOS and chronic inflammation associated with *C. trachomatis* infection [66]. Therefore, due to potential complications, the importance of screening is emphasized, especially in the context of asymptomatic patients who may insidiously develop persistent infections [66]. Some analyses indicate that in the case of persistent *C. trachomatis* infections, 26% of women develop pelvic inflammatory disease (PID), associated with ectopic pregnancy (10%), and in the case of recurrent PID, 38% of women experience infertility (Haggerty et al., 2010; Davies et al., 2017; Tsevat et al., 2017) [67,68,69]. In addition, disruption of the lower genital tract microbiome results in the risk of, for example, bacterial vaginosis (BV), which impairs embryo implantation or fetal growth, or, for example, vaginitis, premature or stillbirth or infertility. Moreover, in women with PCOS, the occurrence of more favorable growth conditions for pathogenic microorganisms, such as atypical pathogens, than those desirable in the vaginal microbiome, such as *Lactobacillus* spp., is explained by the predominance of amino acid metabolism pathways, oxidative phosphorylation and various N-glycan biosynthesis processes [6,60,61,62,63,64,70].

In addition, the role of sex hormones in regulating, for example, the composition of the vaginal microbiome has been indicated, as well as the opposite effect, i.e., the vaginal and intestinal microbiome on sex hormones. Hence, this issue is of particular importance in women with polycystic ovary syndrome (PCOS) [71,72]. Therefore, the importance of a detailed analysis of the microenvironment of the lower genital tract is increasingly emphasized, which would help understand the mechanisms involved in reproduction in patients with PCOS. The study by Tu et al. (2020) of women with PCOS indicates a close relationship between reproductive dysfunction and the length of the menstrual cycle with changes in the genital tract microbiome compared to women without this syndrome [6]. Irregularities in menstrual cycles and abnormalities in hormone levels in women with PCOS cause disturbances in the vaginal microbiome. Based on studies conducted in women with PCOS and on animal models, a relationship has been shown between the syndrome and the dynamic balance of the vaginal microbiome, which differs from the conditions in healthy women and, consequently, leads to dysbiosis. Tu et al. (2020) indicate changes in the genital tract microbiome in patients with PCOS and a reduced share of *Lactobacillus* spp., which favors the development of microorganisms such as *C. trachomatis* and *M. hominis* examined in our study, but also *G. vaginalis* and *Prevotella* [6,22,71].

Some researchers suggest that an abnormal LH:FSH ratio in women with PCOS may result in vaginal dysbiosis due to decreased *Lactobacillus* spp. in the vaginal microbiome [6]. Although the relationship between the vaginal microbiome and hormone levels has not been fully elucidated in PCOS, there are some considerations about the influence of the gut microbiome, sex hormones and the impact on the female reproductive microbiome [5,73]. Disruption of the female reproductive microbiome is associated with symptoms that are particularly common in PCOS patients, such as infertility, miscarriage, preterm birth and low IVF transplant rates [6,63,74,75,76,77]. *Lactobacillus* spp. may be present in women with vaginitis and infection, but detailed analyses have shown that *L. iners* predominates [78,79,80]. However, *L. iners* are characterized by the inability to produce antimicrobial compounds (e.g., hydrogen peroxide, D-lactic acid) [81,82], and in the case of routine vaginal microbiological examinations, such an assessment is not possible. Hence, the results of the assessment of vaginal biocenosis often seem normal, with the presence of *Lactobacillus* spp., but without the awareness that its role is limited [81,82,83]. Currently, there is not much detailed data on the interaction between PCOS and the vaginal microbiome. However, some studies indicate significantly increased levels of “genital mycoplasmas” in patients with PCOS. Therefore, there are even suggestions, as in the study by Hong et al. (2020), to use them as a potential screening biomarker for PCOS, because the number of mycoplasmas in the vaginal microbiome, even in the range of 0.02%, was highly correlated with a high probability of PCOS [5,9,84]. In our study, this percentage is quite significant, as the total number of infections caused by genital *Mycoplasma* spp. and *Ureaplasma* spp. among all (*n* = 490) examined patients amounts to almost 18% and constitutes almost half of the identified atypical pathogens (*n* = 196).

Vaginal dysbiosis in PCOS associated with a reduced proportion of *Lactobacillus* spp. causes an increase in pH, which favors the growth of bacteria such as *G. vaginalis*, and consequently facilitates co-infection with atypical bacteria such as *Mycoplasma* spp., *Ureaplasma* spp., and *C. trachomatis*, leading to pelvic inflammatory disease (PID) and several complications [6,84]. *G. vaginalis*, thus, paves the way for other pathogens, as it degrades vaginal mucin through the production of enzymes such as sialidase and vaginolysin. Therefore, it can be suspected that bacterial vaginosis may occur more frequently in patients with PCOS and/or infertility due to reduced vaginal immunoprotective mechanisms [70,85]. Furthermore, a potential increase in susceptibility to vaginal infections in PCOS may result in increased production of proinflammatory cytokines, leading to ovarian inflammation and, ultimately, to ovarian dysfunction. It has also been suggested that the imbalance of the vaginal microbiome in PCOS plays an important role in the immune homeostasis of the reproductive system, the disruption of which causes a pro-inflammatory state [72,73,86].

Therefore, the next goal of our study was to analyze the possible increase in the immune response in the presence of infection with atypical pathogens. Hence, the selection of control groups played a key role in the evaluation of the proinflammatory cytokines we selected, IL-1β, IL-6 and TNF-α. A group of patients with PCOS who were not infected with atypical pathogens (control group A) and a second group consisting of patients without PCOS and with no infections were selected. The selection of control groups was related to the fact that such proinflammatory cytokines participate in the regulation of energy, lipid and carbohydrate metabolism and their expression in subcutaneous and intraperitoneal adipocytes [87,88,89], regardless of atypical infection. These cytokines correlate with the development of obesity accompanying PCOS syndrome and its consequences, such as type 2 diabetes, hypertension and metabolic disorders. An association of increased IL-6 level with lipid disorders or insulin resistance has also been demonstrated, which translates into hormonal metabolism and some endocrine disorders [90]. In addition, IL-6 and TNF-α affect their secretion levels. Proinflammatory cytosine TNF-α has a wide range of functions both in response to infection, tumor transformation, and in metabolic processes and in the functioning of adipose tissue. Elevated levels of TNF-α in serum and tissues are associated with ongoing infection, inflammation or inflammatory diseases and are responsible for the severity of their course. TNF-α is produced mainly by monocytes/macrophages in inflammatory diseases, but also by, among others, mast cells, T and B lymphocytes, NK cells, neutrophils, endothelial cells, smooth muscle and heart cells, fibroblasts and osteoclasts [90,91]. Hence, TNF-α is believed to play an important role in the normal immune response. However, its abnormal or excessive production can have harmful effects and lead to the development of pathological changes and diseases, including autoimmune ones, e.g., rheumatoid arthritis (RA) [92], inflammatory bowel disease (IBD) [93] and even non-infectious uveitis (NIU) [94]. In the intestine, elastase produced by neutrophils induces matrix degradation, increased by accumulated macrophages producing TNF-α, IL-1 and IL-6, and subsequent epithelial damage, endothelial activation and vascular dysfunction [95]. Hence, although the role of TNF-α in immune function is well established, many mechanisms of action in disease progression require further analysis. In particular, the involvement of TNF-α through its excessive secretion in the activation and accumulation of fibroblasts, resulting, for example, in fibrosis and stricture formation [94].

Our results for the TNF-α analysis in PCOS patients showed a distinct increase of this proinflammatory cytokine in infections with atypical pathogens, and in particular the highest values were observed in the presence of *C. trachomatis* compared to the control groups. Such findings may highlight a unique inflammatory or immunomodulatory effect of the intracellular pathogen *C. trachomatis* on TNF-α expression, potentially directing further studies on pathogen-specific immune pathways and implications for therapeutic interventions (Table 2). In the case of the other proinflammatory cytokines we studied, IL-1β and IL-6, the differences in the induced immune response compared to the control groups were not as notable, although the increase was observed in all infections and, again, with the greatest intensity in the presence of *C. trachomatis* infection. Due to the similar response in the “genital mycoplasmas” of *Mycoplasma* spp. and *Ureaplasma* spp. infections, we analyzed them collectively. It is important to understand the consequences of increased TNF-α cytokine in PCOS. Due to the chronic low-grade inflammation that characterizes this syndrome, elevated TNF-α levels are associated with hyperandrogenism, the development of insulin resistance and obesity because TNF-α interferes with insulin signaling.

The importance of the proinflammatory cytokine TNF-α in the pathophysiology of PCOS is, therefore, emphasized due to its involvement in promoting insulin resistance, hyperandrogenism and follicular development, as well as its association with the development of chronic inflammatory diseases due to TNF-α polymorphisms at the genetic level [96,97,98,99,100]. It should be noted that even though in the presence of PCOS syndrome (control group A) the values of this cytokine were increased compared to the group of completely healthy patients (control group B), infections with atypical pathogens significantly increased the response of the immune system. Of course, it is also worrying that the secretion of this proinflammatory cytokine is observed regardless of the presence of infection, for example with atypical pathogens. Studies by other authors in patients with PCOS have shown the presence of an inflammatory response in the endometrial tissue, the sensitivity of which is enhanced by increased levels of inflammatory factors. This leads to embryo implantation failure and miscarriages in such patients, and then a low pregnancy rate, even despite pharmacological regulation of ovulation. Therefore, some researchers suggest the importance of assessing proinflammatory cytokines, e.g., TNF-α and IL-1 in endometrial secretions as predictors of pregnancy, although the analysis of these factors in blood serum may be more accessible [101,102,103,104].

In the studies of Ha et al. (2022), an analysis of TNF-α concentration in serum and uterine secretions of patients with PCOS was performed [105]. Both biological materials showed increased levels of TNF-α compared to healthy women, both systemically and locally, and confirmed the correlation with obesity, IR and androgen status in these patients [105,106]. Moreover, the production of interleukin-1 (IL-1), interleukin-6 (IL-6) and tumor necrosis factor-alpha (TNF-α) analyzed in this study is also the result of the transition of macrophages from the anti-inflammatory M2 state to the pro-inflammatory M1 state, which is of great importance in women with PCOS, as obesity and IR additionally promote this mechanism [107]. Pro-inflammatory IL-1 is also multifunctional. Its importance in the processes of fertilization and implantation of the fertilized egg in the uterus, as well as in the activation of the hypothalamic–pituitary–adrenal (HPA) axis, is emphasized, thus controlling adrenal steroidogenesis and metabolic factors such as insulin. In vitro studies have also shown that in human granulosa and thecal cells and in small and large follicles, IL-1β stimulates basal progesterone secretion. Moreover, increased levels of IL-1β may also be the result of anovulation in women with PCOS [108,109]. IL-6 and TNF-α levels are significantly higher in patients with metabolic disorders, including women with PCOS, also due to chronic inflammation [110,111,112]. However, although some correlations have been shown between obesity and the secretion of proinflammatory cytokines, many studies currently question these relationships. An example is the study by Mazloomi et al. (2023), which showed that the increase in IL-1 and IL-6 levels in the studied women with PCOS was not dependent on BMI [113]. Therefore, the analysis of cytokines in women with PCOS is additionally complicated by many factors, such as the current chronic inflammation, as the values are usually elevated compared to women without this syndrome. This was also confirmed by the comparison of cytokine production between control group A (women with PCOS without infection) and control group B (women without PCOS and without infection) in the presented work. In our study, elevated values were observed in the presence of atypical pathogens, especially for TNF-α, but also IL-1β and IL-6, although to a lesser extent.

*C. trachomatis* antigens are recognized by epithelial cell receptors, and their activation triggers the immune system response through the release of proinflammatory cytokines such as IL-1β, IL-6 and TNF-α [114,115,116,117] and chemokines that stimulate inflammatory cells. Our study confirms that the production of proinflammatory cytokines such as TNF-α, IL-1 and IL-6 is induced when *C. trachomatis* invades the host reproductive system because the main goal of the immune system is to induce or enhance inflammation against chlamydia [118], then inhibit the growth of these microorganisms and control the infection, as well as prevent or slow down the formation of chlamydial lesions. However, sometimes it can also lead to the development of chronic infection and related complications [40,119]. The importance of induction of TNF-α production in response to the presence of intracellular pathogens, such as chlamydia, and its antibacterial effect is emphasized [120]. In response to chlamydial infection, TNF-α leads to a reduction in chlamydial growth by cooperating with IFN-γ, which results in an increased activity of indoleamine 2,3-dioxygenase (IDO) and inhibition of host cell metabolism. In addition, TNF-α induces apoptosis of host cells, which provides suitable growth conditions for chlamydia, thus influencing their survival. It is also suspected that TNF-α increases phagocytic activity [40,121]. Increased levels of TNF-α were observed in the host serum, which was used in our studies, but were also found in other biological materials, e.g., vaginal secretions. Interestingly, it sometimes happens, even in chlamydial infection, that in the presence of TLR2 or TLR4 deficiency in macrophages, there may be a decrease in TNF-α levels [40,122]. On the other hand, the role of TNF-α in chlamydia clearance in vivo has not been fully elucidated. Animal models suggest that TNF-α is not essential for this process, especially in primary genital infections. Although an increase in TNF-α production has been observed in reproductive tissues, especially in the first week of infection, levels may also gradually decrease during infection [120]. TNF-α stimulation, for example, from other uninfected cells is also a result of IL-1 secretion by intact cells [123]. In addition, vasodilation increased endothelial permeability, activation and influx of neutrophils or increased expression of adhesion molecules may occur as a result of released cytokines. The immune response to infection involving cells such as neutrophils aims to limit the ability of bacteria to multiply and spread the infection [17,124]. However, such an inflammatory response, in addition to its primary goal of eliminating microorganisms from the host, may cause additional effects of tissue damage and scarring [17,117,125]. Hence, in *C. trachomatis* infection, the role of TNF-α in immunopathological damage is also indicated [40]. Elevated TNF-α values in our patients, with a relatively high percentage of *C. trachomatis* infections, may even suggest the presence of chronic infections. This fact is particularly worrying, due to the action of TNF-α not only as a neutrophil chemoattractant, a transcription factor in the NF-κB and MAP kinase pathways, inducing inflammation, immune cell proliferation, apoptosis and fibrosis, but also as a factor contributing to the pathology of the upper genital tract [120,126,127]. Due to its numerous consequences and the need for targeted microbiological tests, *C. trachomatis* infection should become one of the routine gynecological examinations. One of the indications, in the absence of other causes, may be an increase in the number of leukocytes in the microbiological test, which can already be observed in a direct microscopic preparation made from a vaginal smear. Leukocyte exudate due to inflammation is, among other things, the result of the supporting effect of TNF-α. Infections, especially chronic ones, are characterized by numerous complications resulting from interactions at the cellular level of both TNF-α, IL-6 and IL-8. The influence of these cytokines has been demonstrated in connection with tissue fibrosis, scar formation or histopathological changes caused by the acceleration of collagenase release by TNF-α [128]. In the presented study, the concentration of the proinflammatory cytokine IL-6, although increased, was not at the same level as in the case of TNF- α. However, an increase was observed in each infection with an atypical pathogen, although the highest levels were present in patients with *C. trachomatis* infection. A slight increase in our patients could also suggest a persistent infection because, in the case of IL-6, its importance in the defense against infection is emphasized [129]. It controls the infection by mediating inflammation, induces the recruitment of white blood cells and promotes neutrophil apoptosis [24,130]. In the case of *C. trachomatis* infections resulting in tubal infertility, long-term tubal adhesion IL-6 is overexpressed [131,132], but it is also detected, for example, in the serum of asymptomatic patients with chlamydial infection [133]. It is believed that due to the association of IL-6 with inflammatory pathology and fibrosis, it is important to determine the level of this cytokine in patients with tubal factor infertility/tubal adhesions [131,134], which in women with PCOS would be of great importance from the point of view of fertility problems. Moreover, it has been shown that the initial production of IL-6 in response to infection is necessary for the host organism to respond appropriately to the immune system. However, the consequence of persistent, long-term increased levels of this cytokine is local tissue damage and increasingly reported adverse obstetric outcomes, such as recurrent miscarriages [135]. Moreover, chronic inflammation caused by *C. trachomatis* infection and increased production of, e.g., IL-6 result, among others, in the stimulation of cell proliferation and secretion of proangiogenic and immunosuppressive factors, as well as an increased risk of HPV infection or neoplastic changes [136,137,138]. Based on the analyses of Wang et al. (2022) regarding cytokines induced by *C. trachomatis* infection, a member of the IL-6 cytokine family, leukemia inhibitory factor (LIF), was identified [139]. The purpose of this factor production is to maintain epithelial homeostasis and tissue repair, also in *C. trachomatis* infection. However, its long-term expression can lead to harmful epithelial transformations and result, for example, in an increased risk of infertility or ectopic pregnancy. However, also in the case of IL-6 and the pathogenesis of *C. trachomatis* infections in women, its role is not fully explained and requires further analysis. The important role of this interleukin in controlling infections is indicated using anti-IL-6 immunotherapy, which increases the susceptibility of patients to infections [131,140]. IL-6 belongs to the cytokines induced by IL-1β belonging to the IL-1 family. In the case of our group of patients, the levels correlated with IL-6 and were also not very high. This is surprising because the immune system of our patients with PCOS did not undertake a clear “fight” against the ongoing infection. Stimulation of the secretion of proinflammatory cytokines and chemokines such as IL-1β supports the host’s defense against chlamydial infection and participates in the immune response in both acute and chronic infection [141]. Increased IL-1 levels in *C. trachomatis* infection are observed in the cervix and blood or synovial tissue [119,142]. The IL-1 family has been shown to have a variety of effects. It includes not only members regulating inflammation during the progressing infection, but also those whose activity causes immunopathological damage in chlamydial infection, e.g., pathological mechanism of oviduct edema and tissue fibrosis [40,136]. Infections with other atypical pathogens, such as *Mycoplasma* spp. of the genital tract, due to their adherence to epithelial cells, also trigger acute inflammation. Some mycoplasmas can affect host cells at the regulatory and/or functional level, disrupting and altering their cellular pathways. The initial phase of infection involves a nonspecific innate immune response, which plays a key role in host defense. Through Toll-like receptors, innate immune cells (neutrophils, macrophages and NK cells) can recognize pathogen-associated molecular patterns (PAMPs) and kill mycoplasmas [143,144]. Although not all mechanisms are yet fully elucidated from the perspective of the immune response, an increasing number of studies highlight the involvement of genital *Mycoplasma* spp. in infections of the female reproductive tract. As a metabolic product, *M. hominis* produces ammonia leading to oxidative stress and its accompanying effects, such as lipid and protein oxidation, causing damage to some cellular components, DNA, impairment of cellular functions and induction of cell death. It is suggested that the involved pathological processes result in increased levels of proinflammatory cytokines, such as TNF-α, IL-1 and IL-6, among others, in response to infection [42,145]. Also in infections with genital mycoplasmas (*Mycoplasma* spp., *Ureaplasma* spp.) the key role of IL-6 is emphasized, which affects not only the transformation of inflammation from acute to chronic, but also the weakening of the ciliary activity of the fallopian tube epithelium and other proinflammatory cytokines, such as TNF-α and IL-1 beta in the immune response of the organism [86,146,147,148,149,150,151]. Activation of NF-κB with the participation of TLR2/6 and TLR4 in the genital epithelium leads to the production of proinflammatory cytokines, such as IL-6 or TNF-α [152,153]. Moreover, an increase in the level of IL-1β and IL-6 was demonstrated in the study by Marconi et al. (2011) in the case of *M. hominis* in women with intraamniotic infection and premature labor [154]. In genital mycoplasma infections, an increased expression of TLR4 is observed as a result of the induction of autophagy in cells, and the influence of mycoplasmas on increased apoptotic signaling of cells is also indicated [155]. The induction of a proinflammatory response is also the result of changes resulting from cell damage, which affect a whole range of processes regulating apoptosis, oxidative stress or changes in Toll-like receptor genes [156]. Moreover, the study by Massaro et al. (2009) showed that increased IL-6 concentrations in *M. hominis* infection may even be a specific marker of preterm labor [157]. On the other hand, the study by Nicolau et al. (2022) conducted on pregnant women with genital mycoplasma infection (*M. hominis*, *U. urealyticum*, *U. parvum*) shows an increase in various proinflammatory cytokines, but especially IL-1α, IL-1β, IL-6, TNFα and IL-8 [42]. However, the increase in the concentration of these cytokines was quite diverse because the biological material used was diverse: patients’ blood, cervicovaginal fluid, amniotic fluid and umbilical cord blood. In addition, a mild inflammatory reaction was observed in the cervicovaginal space in women infected with genital mycoplasmas. It was suggested that this process may serve to prepare the cervical tissues for an immune response in the event of an attempt to penetrate the amniotic cavity by pathogens [158]. Hence, the influence of inflammatory markers, such as cytokines TNFα and IL-6, on women’s reproductive health is crucial and necessary for effective ovarian function, which is why their role in the case of PCOS is worth emphasizing [156,159,160]. Studies show that women with PCOS are more susceptible to infertility compared to healthy women. In the case of this syndrome, an increase in IL-6 secretion is observed and induced by IL-1 and TNF-α. IL-6 affects the activity of the corpus luteum, fetal development and the production of sex hormones. Hence, its excess adversely affects the fertility of patients [161,162]. Increased levels of TNF-α and IL-6 in women with PCOS are also observed in follicular fluid and serum as a result of a weak intestinal barrier and the penetration of LPS of Gram-negative bacteria into the body. Many studies emphasize the importance of intestinal dysbiosis in women with PCOS, due to the ongoing chronic inflammation caused by endotoxemia [163,164,165,166]. The complication of chronic inflammation in the ovaries in women with PCOS results in insulin resistance, increased levels of androgens LH and then testosterone and abdominal obesity. The synthesis of sex hormones is also limited [167,168]. This emphasizes the importance of the intestinal microbiota in the development of chronic inflammation in the course of PCOS. Studies confirm the participation of proinflammatory cytokines in embryonic and reproductive development [169]. Hence, their increase in periovarian adipose tissue was observed in studies on animal models of PCOS [160,170]. In women with PCOS, many studies have been conducted on the gut microbiome, while research on the vaginal microbiome and its role in PCOS is still rather scarce. However, it has been suggested that disruption of the genital microbiome may potentially affect hormonal balance [9] and promote the development of pathogenic microorganisms such as “genital mycoplasmas” [5] or *G. vaginalis* [70]. It should be emphasized that the causes of infertility in patients with PCOS may be very diverse [171], including the result of a constantly present disease and chronic inflammation, which is reflected in the results of biomarkers, e.g., proinflammatory cytokines, chemokines and CRP protein. Additionally, in these patients, infections with atypical pathogens may develop more easily and insidiously. Especially in the case of genital mycoplasmas, which on the one hand can only be considered potentially pathogenic, on the other hand, infections can result in numerous complications, e.g., in the genital tract. Hence, based on our studies, not all proinflammatory cytokines showed a significant increase compared to the control group A of patients diagnosed with PCOS and healthy patients without PCOS. Therefore, some caution should be exercised in using such biomarkers as predictors of PCOS or atypical infections. However, it is worth emphasizing that omitting tests for atypical pathogens in women with PCOS may additionally deepen reproductive difficulties and intensify the already existing chronic inflammation. In fact, already at the stage of examining the vaginal biocenosis in women with PCOS, attention should be paid to such parameters as even a slight increase in pH, a slight elimination of *Lactobacillus* spp., the presence of *G. vaginalis* or the diagnosis of bacterial vaginosis (BV) because, as studies show, such a microenvironment favors the development of atypical microorganisms, especially of the genital Mycoplasma and Ureaplasma genera, and paves the way for many other sexually transmitted pathogens, such as *C. trachomatis* [5,6,70].

Another problem is the fact that these infections can often be asymptomatic, and patients will not report any alarming problems. However, in the case of PCOS, especially in the group of women of reproductive age, despite possible doubts about screening tests for atypical pathogens, it is worth introducing targeted microbiological tests as an element of routine diagnostics in this group of patients, among others due to targeted treatment and prevention of infertility.

## 5. Conclusions

It seems that atypical pathogens, such as intracellular *C. trachomatis* and extracellular, cell wall-deprived *M. hominis* and *Ureaplasma* spp., may play an important role in infections in patients with PCOS. The presence of such pathogens was found in 40% of the examined women with PCOS syndrome, but there was no dominance in the frequency of occurrence between *C. trachomatis* and “genital mycoplasmas” (*M. hominis* and *Ureaplasma* spp.). In infections with atypical pathogens in patients with PCOS, no characteristic clinical features were observed, except for indications in the form of an increase in the number of leukocytes in the assessment of the vaginal biocenosis, suggesting cervicitis, reproductive failure or lower abdominal pain. An additional problem is the inability to detect the presence of atypical pathogens in routine microbiological tests, therefore, confirmation of such etiology requires referral of the patient for additional tests. Therefore, especially in the case of women with PCOS, it is worth considering including such tests as a standard action. Additionally, invasion of host cells by atypical pathogens, such as *C. trachomatis* and infection with “genital mycoplasmas”, can disrupt their functioning and lead to several complications, including infertility. The immune response with the production of proinflammatory cytokines, such as TNF-α, IL-1β and IL-6 observed in response to infection with *C. trachomatis*, but also *M. hominis* and *Ureaplasma* spp., is aimed at inducing or amplifying inflammation by activating immune cells or controlling infection, but can also lead to the survival of microorganisms and irreversible tissue damage in the fallopian tubes [40,122,172,173]. It seems that especially in the case of the proinflammatory cytokine TNF-α there is a close correlation with atypical pathogen infections and a clear response from the immune system, with lower but always elevated values for IL-1β and IL-6 compared to the absence of infection (both in the presence of PCOS and in its absence). These data may suggest, in our analyzed group of patients with PCOS, the persistence of atypical infections and may be a starting point for future improved diagnostics and further studies in this area.

## Figures and Tables

**Table 2:** Welch test: Two-sample assumption of unequal variances for TNF-α. CHLAM—patients with PCOS and *C. trachomatis* infection; MU—patients with PCOS and *M. hominis* and *U. urealyticum*/*U. parvum* infection; MUC—patients with PCOS and *C. trachomatis*, *M. hominis* and *U. urealyticum*/*U. parvum* infection; control with PCOS and without infection—control group A; control without PCOS and without infection—control group B.

TNF-α	Mean Concentration Values ± SD	*p*
*CHLAM*	26.27 ± 6.50	<0.05
*CONTROL WITH PCOS (GR.A)*	8.02 ± 3.53	
*MU*	22.16 ± 5.29	<0.05
*CONTROL WITH PCOS (GR.A)*	8.02 ± 3.53	
*MUC*	29.90 ± 7.32	<0.05
*CONTROL WITH PCOS (GR.A)*	8.02 ± 3.53	
*CHLAM*	26.27 ± 6.50	<0.05
*MU*	22.16 ± 5.29	
*CHLAM*	26.27 ± 6.50	>0.05
*MUC*	29.90 ± 7.32	
*MU*	22.16 ± 5.29	<0.05
*MUC*	29.90 ± 7.32	
*CONTROL WITH PCOS (GR.A)*	8.02 ± 3.53	<0.05
*CONTROL WITHOUT PCOS (GR.B)*	5.24 ± 1.30	

**Table 3:** Welch test: Two-sample assumption of unequal variances for IL-1β. CHLAM—patients with PCOS and *C. trachomatis* infection; MU—patients with PCOS and *M. hominis* and *U. urealyticum*/*U. parvum* infection; MUC—patients with PCOS and *C. trachomatis*, *M. hominis* and *U. urealyticum*/*U. parvum* infection; control with PCOS and without infection—control group A; control without PCOS and without infection—control group B.

IL-1β	Mean Concentration Values ± SD	*p*
*CHLAM*	3.07 ± 0.82	<0.05
*control with PCOS (gr. A)*	1.33 ± 0.39	
*MU*	2.47 ± 0.73	<0.05
*control with PCOS (gr. A)*	1.33 ± 0.39	
*MUC*	3.56 ± 1.25	<0.05
*control with PCOS (gr. A)*	1.33 ± 0.39	
*CHLAM*	3.07 ± 0.82	<0.05
*MU*	2.47 ± 0.73	
*CHLAM*	3.07 ± 0.82	>0.05
*MUC*	3.56 ± 1.25	
*MU*	2.47 ± 0.73	<0.05
*MUC*	3.56 ± 1.25	
*control with PCOS (gr. A)*	1.33 ± 0.39	<0.05
*control without PCOS (gr. B)*	1.14 ± 0.18	

**Table 4:** Welch test: Two-sample assumption of unequal variances for IL-6. CHLAM—patients with PCOS and *C. trachomatis* infection; MU—patients with PCOS and *M. hominis* and *U. urealyticum*/*U. parvum* infection; MUC—patients with PCOS and *C. trachomatis*, *M. hominis* and *U. urealyticum*/*U. parvum* infection; control with PCOS and without infection—control group A; control without PCOS and without infection—control group B.

IL-6	Mean Concentration Values ± SD	*p*
*CHLAM*	2.77 ± 0.80	<0.05
*CONTROL WITH PCOS (GR.A)*	1.61 ± 0.41	
*MU*	2.39 ± 0.62	<0.05
*CONTROL WITH PCOS (GR.A)*	1.61 ± 0.41	
*MUC*	3.27 ± 1.04	<0.05
*CONTROL WITH PCOS (GR.A)*	1.61 ± 0.41	
*CHLAM*	2.77 ± 0.80	<0.05
*MU*	2.39 ± 0.62	
*CHLAM*	2.77 ± 0.80	>0.05
*MUC*	3.27 ± 1.04	
*MU*	2.39 ± 0.62	<0.05
*MUC*	3.27 ± 1.04	
*CONTROL WITH PCOS (GR.A)*	1.61 ± 0.41	<0.05
*CONTROL WITHOUT PCOS (GR.B)*	1.25 ± 0.31	

## Data Availability

The original contributions presented in this study are included in the article. Further inquiries can be directed to the corresponding author.
